# MnO_2_-Incorporated Magnetic Nanoparticles with pH-Responsive Drug Release Enhance Hypoxia-Resistant Photodynamic Therapy for Melanoma

**DOI:** 10.3390/pharmaceutics18050586

**Published:** 2026-05-09

**Authors:** Encheng Tian, Yunchang Zhang, Runsheng Wang, Haohan Wu, Mingjian Sun, Yingyi Yan, Lan She, Zhiqiang Ma

**Affiliations:** 1 Graduate School, Naval Medical University, 41 Zhengtong Road, Shanghai 200433, China; tecmant@163.com (E.T.); mingjiansun0308@outlook.com (M.S.); 2 School of Pharmacy, Naval Medical University, 325 Guohe Road, Shanghai 200433, China; yunchangzhang@hotmail.com (Y.Z.); wrs19353960676@163.com (R.W.);

**Keywords:** photodynamic therapy, drug delivery system, pH-responsive, oxygen enhancement, oxidative stress

## Abstract

**Background/Objectives:** Photodynamic therapy (PDT) is severely limited by the hypoxic tumor microenvironment, which restricts reactive oxygen species (ROS) generation and compromises therapeutic efficacy. To address this critical barrier, we engineered a multifunctional nanocomposite (Pha@FSMP) integrating oxygen supplementation, pH-responsive drug release, and magnetic targeting for enhanced PDT. **Methods:** The platform is constructed with a superparamagnetic Fe_3_O_4_ core, coated in amino-functionalized mesoporous silica (mSiO_2_) loaded with MnO_2_ as an oxygen-evolving catalyst, and surface-conjugated with the pH-responsive copolymer PEG-*b*-PAsp to encapsulate the hydrophobic photosensitizer Pha. We characterized its core physicochemical and functional properties, and evaluated its photodynamic efficacy via in vitro cellular assays and in vivo studies in a murine melanoma model. **Results:** In vitro assays demonstrated significant elevation of intracellular ROS levels and enhanced PDT-mediated cytotoxicity against B16-F10 melanoma cells. In vivo studies in a murine melanoma model confirmed potent tumor growth inhibition, metastasis suppression, and prolonged survival, accompanied by excellent biosafety. **Conclusions:** Collectively, this oxygen-augmented nanocomposite represents a promising strategy to overcome hypoxia-associated PDT resistance, offering a translatable platform for improved cancer therapy.

## 1. Introduction

Malignant tumors stand as one of the most formidable threats to human health, and a range of therapeutic modalities have been developed for their management, including surgery, radiotherapy, and chemotherapy [1].

However, these approaches are currently associated with drawbacks such as poor targeting ability, significant side effects, and high toxicity [2]. In addition, surgical interventions may impede patient recovery. Given these challenges of cancer treatment, photodynamic therapy (PDT), which originated in the 20th century, has gained prominence [3,4]. PDT utilizes the photodynamic reactions of photosensitizers to produce cytotoxic substances that specially target tumor tissues, achieving therapeutic effects. PDT minimizes side effects and selectively eliminates tumor cells and spares normal tissue. Consequently, PDT has attracted considerable attention in global cancer prevention and treatment research. PDT exerts its anti-tumor effect through three main mechanisms: first, generation of reactive oxygen species (ROS), particularly singlet oxygen, which induces apoptosis or necrosis of tumor cells [5,6]; second, activation of systemic anti-tumor immune responses [7]; and third, disruption of the tumor-associated vascular systems, leading to ischemic death of tumor cells [8]. These mechanisms play pivotal roles in long-term tumor control and treatment. PDT consists of three key components: photosensitizers, light sources and molecular oxygen [9]. A fundamental limitation of conventional PDT is the insufficient selectivity and affinity of photosensitizers for target lesions, which is further exacerbated by the inconsistent metabolic processes of photosensitizers in tumor tissues [10]. This often results in suboptimal accumulation of photosensitizers. Moreover, the hypoxic microenvironment commonly present in solid tumors severely restricts the generation of cytotoxic ROS, significantly impairing the therapeutic efficacy of PDT [11].

To overcome these challenges, researchers have turned to combination therapies, especially the integration of PDT with chemotherapy [12]. The rationale for this strategy lies in the synergistic effects between the two modalities: PDT can enhance tumor vascular permeability and improve drug delivery, while certain chemotherapeutic agents can sensitize cancer cells to ROS-induced damage. This approach not only improves therapeutic efficacy but also enables dose reduction of both photosensitizers and chemotherapeutic drugs, potentially reducing systemic toxicity and addressing drug resistance [13,14,15].

In this context, nanoparticle-based delivery systems have significant advantages over traditional organic small-molecule drugs. Nanoparticles can be engineered to co-deliver both photosensitizers and chemotherapeutic agents directly to tumor tissues by leveraging the enhanced permeability and retention (EPR) effect. Furthermore, smart nanocarriers can be designed to respond to specific tumor microenvironment stimuli (such as pH, enzymes, or redox conditions) or external triggers (such as light) for controlled drug release. Some advanced systems can even modulate the tumor hypoxic microenvironment by catalyzing the conversion of endogenous hydrogen peroxide (H_2_O_2_) to oxygen, thereby potentiating PDT through continuous oxygen supply and promoting ROS generation under irradiation [16]. These multifunctional properties make nanoplatforms promising carriers for achieving more effective and precise cancer therapy.

Previous studies have reported that the rapid proliferation of malignant tumor cells produces substantial amounts of lactic acid and H_2_O_2_ in the tumor microenvironment. A large accumulation of lactic acid leads to a lower pH at the tumor site compared to normal human tissues, with a pH of approximately 5.2. It is well known that H_2_O_2_ can be catalytically decomposed into O_2_ by MnO_2_.The favorable biocompatibility of MnO_2_ at low doses enables sustained and effective implementation of PDT [17].

**Figure 1:**
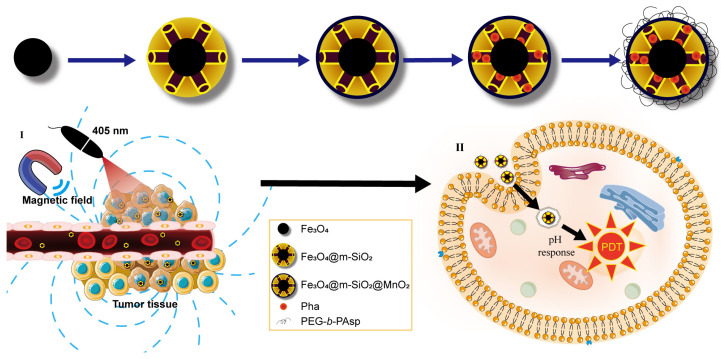
Schematic illustration of the pH-responsive and magnetically targeted drug delivery system for photodynamic cancer therapy. (**I**) Laser irradiation on the tumor microenvironment; (**II**) schematic diagram of cellular uptake and intracellular drug release.

To address the hypoxic limitation of photodynamic therapy (PDT), we engineered a multifunctional MnO_2_-based nanocomposite delivery system (Pha@FSMP) designed to elevate intratumoral reactive oxygen species (ROS) generation (Figure 1). The system is constructed via a stepwise assembly: superparamagnetic Fe_3_O_4_ cores are first coated with amino-functionalized mesoporous silica (mSiO_2_) to enable magnetic targeting, followed by loading MnO_2_ into the mesopores to catalyze oxygen production. The nanoparticle surface is then grafted with the pH-responsive polymer PEG-*b*-PAsp (polyethylene glycol-b-poly (aspartic acid)) [18], and further self-assembled with the photosensitizer Pha (a chemically stable, representative photosensitizer). This integrated platform leverages magnetic targeting for tumor accumulation, MnO_2_-mediated oxygenation to overcome hypoxia, and pH-responsive release of Pha to enhance local drug concentration. Collectively, this nanocomposite aims to amplify PDT efficacy by boosting ROS generation at the tumor site, while simultaneously minimizing systemic photosensitizer toxicity.

## 2. Materials and Methods

### 2.1. Reagents

Ferric chloride hexahydrate (FeCl_3_·6H_2_O), sodium acetate, Phoeophorbide a (Pha), and sodium citrate tribasic dihydrate (Na_3_Cit·2H_2_O) were purchased from Adamas Industrial Inc. (Shanghai, China), and methoxy polyethylene glycol amine (CH_3_O-PEG-NH_2_, MW = 5000) was purchased from Aladdin Industrial Corporation (Shanghai, China). N-hydroxysuccinimide (NHS) and carbodiimide crosslinkers (EDC) were purchased from Aladdin, while sodium dodecylbenzene sulfonate (SDBS) and cell apoptosis detection kits (Annexin V-FITC/PI) were supplied by BD Biosciences (San Jose, CA, USA). Cell viability was assessed using Cell Counting Kit-8 (CCK-8, Beyotime Biotechnology, Shanghai, China, Cat# C0037).

### 2.2. Cell Culture

Murine melanoma B16-F10 cells (certified by the National Key Laboratory of Medical Immunology, Shanghai, China, Cell ID: IMM-B16-2020-011) were cultured in RPMI-1640 medium (Gibco, Shanghai, China, Cat# 31800022) supplemented with 10% fetal bovine serum (FBS, Gibco, Shanghai, China, Cat# 10099141) and 1% penicillin-streptomycin (Gibco, Shanghai, China, Cat# 15140122). All cells were incubated at 37 °C in a humidified 5% CO_2_ atmosphere.

### 2.3. Fabrication of Nanocomposites

#### 2.3.1. Constitutes of Carrier Materials and Model Drug

Phoeophorbide a (Pha) was selected as the model photosensitizer for its high stability and reliable experimental reproducibility. It retains the fundamental structure of second-generation porphyrin-based photosensitizers and is widely used as a model drug in PDT. Furthermore, PEG-*b*-PAsp, a pH-responsive amphiphilic copolymer, was utilized as the drug carrier, given its well-documented pH responsiveness that was validated by preliminary experiments [19].

#### 2.3.2. Design Strategy of Nanocomposites

The nanocomposites were synthesized using superparamagnetic iron oxide nanoparticles (SPIONs) as the magnetic core. Mesoporous silica nanoparticles (MSNs) served as the photosensitizer carrier. Through two key modifications—first, immobilizing manganese dioxide (MnO_2_) to catalyze the generation of reactive oxygen species (ROS), and second, introducing polymer modification—multifunctional drug-loaded nanocomposites were successfully prepared.

### 2.4. Preparation of Nanocomposites

#### 2.4.1. Synthesis of Fe_3_O_4_ Nanoparticles

Fe_3_O_4_ nanoparticles were synthesized through a solvothermal method. Initially, 1.44 g of Na_3_Cit were dissolved in 180 mL of ethylene glycol under continuous stirring until complete dissolution. Subsequently, 3.3 g of FeCl_3_·6H_2_O and 7.26 g of sodium acetate were sequentially added and fully dissolved. This solution was then sealed within a hydrothermal reactor and reacted at 200 °C for 10 h. After cooling to room temperature, magnetic separation was employed, yielding a precipitate that underwent three wash cycles with anhydrous ethanol and deionized water, aided by ultrasonication. The washed nanoparticles were further dried in a vacuum drying oven (Shanghai Hecheng Instrument Manufacturing Co., Ltd., Shanghai, China) at 50 °C overnight.

#### 2.4.2. Preparation of Fe_3_O_4_@mSiO_2_

To initiate the preparation of Fe_3_O_4_@mSiO_2_ nanocomposites, 4 mg of Fe_3_O_4_ nanopowder was added into a three-necked flask (Titan, Shanghai, China). Subsequently, 25 mL of deionized water was added for ultrasonic dispersion to obtain a homogeneous suspension. Next, a solution of 1 g cetyltrimethylammonium bromide (CTAB) in a mixture of 25 mL water and 10 mL anhydrous ethanol was stirred for 5 min to ensure complete homogenization. Then, 1 mL of ammonia solution (25–28 wt%) was added and stirred, followed by a gradual dropwise addition of a tetraethyl orthosilicate (TEOS) ethanol solution (5 mL of TEOS dissolved in 15 mL of anhydrous ethanol) with continuous stirring. The reaction was then conducted in an 80 °C oil bath for 2 h. After the reaction, the product was collected by magnetic separation and washed three times alternately with anhydrous ethanol and deionized water. Subsequently, the product was dispersed in 50 mL of anhydrous ethanol and refluxed at 92 °C for 72 h. Upon completion of reflux, magnetic separation and triple washing with anhydrous ethanol and deionized water were performed again. Finally, the product was dried overnight in a vacuum oven at 50 °C.

#### 2.4.3. Preparation of Fe_3_O_4_@mSiO_2_@MnO_2_

For the preparation of Fe_3_O_4_@mSiO_2_@MnO_2_ (FSM), 200 mg of Fe_3_O_4_@mSiO_2_ was dispersed in 10 mL of deionized water with gentle stirring. After 5 min of stirring, 0.036 g of MnCl_2_·4H_2_O was added to the dispersion. A solution of 0.04 g NaOH dissolved in 10 mL of deionized water (0.1 mol/L) was then gradually added dropwise to the mixture to adjust the pH to 9. Stirring was maintained throughout the process, and the reaction was allowed to proceed for 30 min at room temperature. After the reaction, the product was isolated by magnetic separation, and washed three times with deionized water to remove unreacted reagents and byproducts. The washed product was eventually vacuum-dried at 50 °C.

#### 2.4.4. Synthesis of PEG-*b*-PAsp [20]

(a) Synthesis of L-Aspartic Acid Benzyl Ester-N-Carboxylic Anhydride (BLA-NCA)

The first step involved mixing 4.8 g of benzyl L-aspartate (BLA) with 60 mL of anhydrous tetrahydrofuran (THF) in an electromagnetic stirring flask. After heating the mixture to 60 °C, 6 g of triphosgene was added while partially opening the flask cap to release the generated gases. Once the reaction solution turned clear and pale yellow, heating was stopped. Nitrogen (N_2_) was purged through the reaction mixture to remove excess phosgene and HCl gas. The reaction mixture was then concentrated by rotary evaporation to a viscous state. The concentrated solution was diluted with chloroform and gradually added dropwise to 400 mL of excess n-hexane under stirring. The resulting mixture was refrigerated at 4 °C for 12 h to induce precipitation. The crude BLA-NCA product was obtained by vacuum filtration. The crude BLA-NCA product was dissolved in 200 mL of ethyl acetate and then recrystallized with n-hexane. Finally, a light-yellow powder product was obtained after vacuum filtration and drying.

(b) Synthesis of Polyethylene Glycol-*b*-Polybenzyl L-Aspartate (PEG-*b*-PBLA)

0.5 g PEG-NH_2_ was added to 40 mL of anhydrous CH_2_Cl_2_ dried over MgSO_4_. After complete dissolution, 2.5 g of BLA-NCA was added and dissolved. Nitrogen (N_2_) was purged for 10 min to create an inert atmosphere, followed by magnetic stirring at 32 °C for 72 h. The reaction solution was then concentrated to approximately 10 mL at 4 °C, and precipitated with 100 mL of diethyl ether. After settling for 12 h, the precipitate was collected by vacuum filtration and dried to obtain a white PEG-*b*-PBLA powder.

(c) Hydrolysis of PEG-*b*-PBLA

3 g PEG-*b*-PBLA was added to a mixture of 75 mL of 1M NaOH and 75 mL of tetrahydrofuran (THF), and magnetically stirred for 10 h. The reaction solution was concentrated to approximately 10 mL, and dialysis with 0.1M HCl was performed for 24 h, followed by additional dialysis against deionized water for another 24 h. A white, fluffy PEG-*b*-PAsp product was obtained via freeze-drying of the solution.

#### 2.4.5. Fabrication of Fe_3_O_4_@mSiO_2_@MnO_2_/Pha@PEG-*b*-PAsp (Pha@FSMP) Nanocomposites

50 mg of FSM mixture was mixed with an ethanol solution of APTES (30 μL APTES in 20 mL anhydrous ethanol) for 48 h. Subsequently, magnetic separation was conducted, followed by three washing cycles with deionized water and anhydrous ethanol under ultrasonication. Under dark conditions, a THF solution containing 5 mg of Pha was added and stirred until the THF evaporated completely. After magnetic separation, the product was washed three times with deionized water and vacuum-dried; then polymer modification was carried out as follows. Briefly, 20 mg of FSM-NH_2_ was placed in a round-bottom flask with 10 mL of deionized water. After thorough dispersion via ultrasonication, 16 mg of EDC·HCl and 4 mg of NHS were added, and the mixture was stirred for 5 min. Then, 50 mg of PEG-*b*-PAsp was added and stirred for 4 h. The resulting composite was freeze-dried to obtain the final Pha@FSMP nanocomposites as a black powder.

### 2.5. Characterization of Pha@FSMP

#### 2.5.1. Morphology and Hydrodynamic Properties

Transmission electron microscopy (TEM; TecnaiG^2^ F20 S-Twin, FEI, Hillsboro, OR, USA; accelerating voltage: 200 kV) was used to observe the morphology. Briefly, 5 mg each of Fe_3_O_4_, Fe_3_O_4_@mSiO_2_, FSM, and Pha@FSMP was dispersed in deionized water in 5 mL centrifuge tubes. Subsequently, 100 μL of each dispersion was drop-cast onto a copper grid, air-dried at room temperature, and subjected to TEM observation.

A Fourier-transform infrared spectrophotometer (FTIR; Nexus Model470, Nicolet, Madison, WI, USA) was employed to characterize the molecular composition and interactions of Pha@FSMP and the control samples (Fe_3_O_4_@mSiO_2_, FSM). Briefly, the nanoparticles were well dispersed in a 5 mL deionized water and vortexed for 5 min.

A Malvern particle size analyzer (ZEN3600, Malvern Panalytical, Malvern, Worcestershire, UK) was used to determine the hydrodynamic size distribution.

The Tyndall effect assay was performed to verify the colloidal dispersion state and stability of Pha@FSMP. Briefly, 0.2 mg of photosensitizer-loaded composite Pha@FSMP was dispersed in 2 mL of phosphate buffered saline (PBS). For the control group, excess free Pha was added into 2 mL of PBS, vortexed thoroughly, and centrifuged to completely remove undissolved precipitates, with the supernatant collected as the saturated PBS solution of free Pha. A 650 nm semiconductor laser was used to irradiate the dispersion in a dark environment, and the Tyndall effect was observed and photographed.

#### 2.5.2. Magnetic Targeting Capability of Pha@FSMP

Magnetic targeting capability of the composite was evaluated using three cuvettes. Specifically, the three cuvettes were respectively loaded with 2 mL of dimethyl sulfoxide (DMSO) (blank control), 2 mL of DMSO containing 1 mg of free photosensitizer (sample 1), and 2 mL of DMSO dispersion of 5 mg photosensitizer-loaded composite (sample 2). All samples were gently mixed to ensure homogeneous dispersion. A neodymium, iron, and boron magnet (NIB, size 3 × 1.5 × 1 cm, purchased from Dongguan Fuqiang Magnetic Industry Co., Ltd., Dongguan, China) was then placed adjacent to the side of each cuvette, and the samples were allowed to stand for a predetermined time. The adsorption phenomena (e.g., material aggregation at the magnet-facing side) were photographed to intuitively assess the magnetic targeting performance of the composite.

#### 2.5.3. Determination of Drug Loading Rate

A standard curve was established to determine the drug loading efficiency of Pha. Briefly, 1 mg of Pha was accurately weighed and dissolved in 1 mL of dimethyl sulfoxide (DMSO), followed by serial proportional dilution. Subsequently, 100 μL of each concentration gradient solution was added to a 96-well plate (Nest, Wuxi, China, Cat. #703001), with three technical replicates per concentration. The absorbance at 405 nm was measured using an ELISA reader (ELx800, BioTek, Winooski, VT, USA), and a standard curve was constructed within the linear range. For the determination of drug loading rate: 1 mg of lyophilized Pha-loaded composite was dispersed in 1 mL of DMSO via ultrasonication for 30 min. After magnetic separation of the mixture, the absorbance (OD value) of the supernatant was measured at 405 nm. The drug loading amount and rate were calculated using the previously established Pha standard curve.

#### 2.5.4. pH-Responsiveness

A total of 3 mg of lyophilized composite was separately dispersed in 10 mL of phosphate-buffered saline (PBS) solutions at pH 5.2 and pH 7.4. Each dispersion was transferred to a dialysis bag (molecular weight cutoff, MWCO: 10 kDa, Viskase Corporation, Darien, IL, USA), which was then dialyzed against 70 mL of pre-equilibrated release medium (pH 5.2 or pH 7.4, respectively). The release medium consisted of PBS, Tween 20, and DMSO at a volume ratio of 98:1:1. All systems were gently stirred under dark conditions and sampled at predetermined time intervals (0.5 h, 1 h, 2 h, 4 h, 8 h, 12 h, 24 h, 48 h). After each sampling, 70 mL of fresh release medium with the corresponding pH was replenished. The cumulative release amount of Pha was determined by measuring the absorbance at 405 nm using an ELISA reader (ELx800, BioTek, Winooski, VT, USA).

### 2.6. Anti-Tumor Effects In Vitro

#### 2.6.1. Cellular Uptake and Distribution of Pha@FSMP

B16-F10 cells in the logarithmic growth phase were adjusted to a concentration of 1 × 10^5^ cells/mL and seeded into two 6-well plates (Nest, Cat. #701001). After incubation for 24 h to allow cell adhesion, the culture medium was aspirated and replaced with fresh medium containing either 0.5 μg/mL of the photosensitizer-loaded composite or 0.25 μg/mL of free Pha. At predetermined time points (0.5 h, 2 h, 4 h, 6 h, 8 h), the cells were rinsed with phosphate-buffered saline (PBS) (pH 7.4), fixed with 4% paraformaldehyde for 15 min at room temperature, and stained as per standard protocols. Cellular uptake efficiency and intracellular distribution of Pha were observed and analyzed using a confocal laser scanning microscope (CLSM; LSM 710, Carl Zeiss, Jena, Thuringia, Germany) with excitation wavelengths of 405/488/633 nm.

#### 2.6.2. Subcellular Localization

B16-F10 cells in the exponential growth phase were adjusted to 5.0 × 10^4^ cells/mL and seeded into 6-well confocal dishes for overnight adhesion. After removing the medium, dishes A, B, and C were treated with 2 mL fresh medium containing 1 μg/mL Pha-loaded composite, while dishes D, E, and F received 2 mL medium with 0.13 μg/mL free Pha. All dishes were incubated for 24 h. DMSO was added at a final concentration of 1% to solubilize the drugs.

After medium removal, dishes A and D were rinsed with PBS (pH 7.4) and incubated with LysoTracker Green (50 nM) for 1 h. Dishes B and E were rinsed and incubated with MitoTracker Green (50 nM) for 45 min. Dishes C and F were rinsed and incubated with Golgi Green for 30 min.

All cells were rinsed with PBS, fixed with 4% paraformaldehyde for 15 min, and stained with DAPI for 3–5 min. Unbound DAPI was removed by rinsing, and cells were resuspended in PBS for confocal laser scanning microscopy (CLSM). Fluorescence emissions were: DAPI-stained nuclei (blue, 461 nm); intracellular free Pha and Pha-loaded composite (red, 660 nm).

#### 2.6.3. Photodynamic Effect In Vitro

In separate 96-well plates, B16 cells in the exponential growth phase were seeded at a density of 1 × 10^5^ cells/mL and divided into groups with or without light irradiation with a wavelength of 660 nm and a power density of 25 mW/cm^2^ for 378 s (a total light dose of 9.45 J/cm^2^; MW-YGX-660/5000 m, Changchun Leishi Optoelectronics Technology Co., Ltd., Changchun, China). After 24 h of incubation, the medium was replaced with fresh medium. Both groups were then treated with a serial dilution (0.78, 1.56, 3.25, 6.25, 12.50, 25.00, 50.00, and 100.00 μmol, respectively) of free Pha and Pha-loaded composite. After 4 h of incubation, the cells were washed, and cell viability was evaluated using the CCK-8 assay.

#### 2.6.4. ROS Generation Analysis

B16-F10 cells in the exponential growth phase were seeded into separate 96-well plates at a density of 1 × 10^4^ cells per well and incubated for 24 h. The medium was then replaced with fresh medium, and serial concentrations (0.6, 1.2, 2.5 μM) of Pha, FSM, and Pha@FSMP were added to the wells with cells treated with an equal volume of normal saline set as the negative control (control group), and a separate group of cells incubated with Rosup (100 μM) to serve as the positive control. After an additional 24 h of incubation, unabsorbed materials were removed by rinsing the cells with phosphate-buffered saline (PBS) (pH 7.4). Subsequently, DCFH-DA reagent was added, and the plates were incubated for another 30 min at 37 °C in the dark. Cells were then irradiated with a 660 nm laser at a total light dose of 9.45 J/cm^2^ (25 mW/cm^2^, 378 s). After laser irradiation, the fluorescence intensity was measured using a multifunctional microplate reader (FLx800, BioTek, Winooski, VT, USA) at an excitation wavelength of 488 nm and an emission wavelength of 525 nm to assess intracellular ROS generation to assess intracellular ROS generation.

### 2.7. Anti-Tumor Effects In Vivo

C57BL/6J male mice (18–20 g, body weight) were purchased from Shrek Animal Co., Ltd. (Shanghai, China). All animal experiments were performed in compliance with the Guiding Principles for the Care and Use of Laboratory Animals, Naval Military Medical University, People’s Republic of China. Protocols were approved by the Institutional Animal Care and Use Committee of the Naval Military Medical University. To establish a subcutaneous melanoma model, 100 μL of culture medium containing 1 × 10^5^ B16-F10 cells was subcutaneously injected into the right hind limb of each mouse, whose tumor sites were bound closely to the NIB magnet. When the tumor volume reached approximately 80 mm^3^, the mice were randomly allocated into six experimental groups to minimize potential confounders (*n* = 5 per group). The sample size of five animals per group was chosen based on extensively published precedent in similar experimental models. The grouping was as follows: saline (negative control), Pha@FSMP + laser irradiation, Pha@ FSMP − laser treatment, free Pha + laser irradiation, free Pha − laser treatment, and FSMP alone. Notably, the dosage of Pha was maintained at 2 mg/kg across all Pha-containing groups. To ensure blinding, group allocation was determined by co-investigators who were not involved in animal handling, dosing, or data collection.

PDT was conducted as following protocols: for the phototherapy groups, the tumor areas were irradiated with a 660 nm diode laser (MW-YGX-660/5000 m, Changchun Leishi Optoelectronics Technology Co., Ltd., Changchun, China) at 45 min post tail vein injection of the respective formulations. The Pha dose was standardized at 2 mg/kg across all groups. The laser parameters were set as follows: power density of 300 mW/cm^2^, irradiation time of 360 s, and total light dose of 108 J/cm^2^. Antitumor efficacy was primarily evaluated based on two parameters: tumor volume measurement and survival rate monitoring of tumor-bearing mice. Throughout the experiment, all mice had ad libitum access to food and water. Tumor size was measured every other day and calculated using the following formula:

$$V=L_{\mathrm{length}}\times L_{\mathrm{width}}^{2}\times 0.5$$

where L_length_ denotes the longest diameter and L_width_ stands for the shortest diameter perpendicular.

In order to investigate the metastasis of tumors in each group, at the end of the experiment, the first mouse that succumbed to the tumor in each group was subjected to necropsy. During necropsy, intact lungs were carefully harvested and immediately fixed in 4% paraformaldehyde solution. Surface metastatic nodules on the lungs were counted under a dissecting microscope. Subsequently, lung tissues were paraffin-embedded, sectioned into thin slices (5 μm, typical thickness) using a microtome, and finally stained with hematoxylin and eosin (H&E) following standard protocols. Histological analysis was performed using an optical microscope (Olympus CKX41-A22PHP, Tokyo, Japan).

### 2.8. Statistical Analyses

The data were shown as mean ± standard deviation. For multiple group analyses, a one-way analysis of variance (ANOVA) was conducted, followed by Tukey’s post hoc test.

## 3. Results and Discussion

### 3.1. Characteristics and Properties of Three Nanoparticles

**Figure 2:**
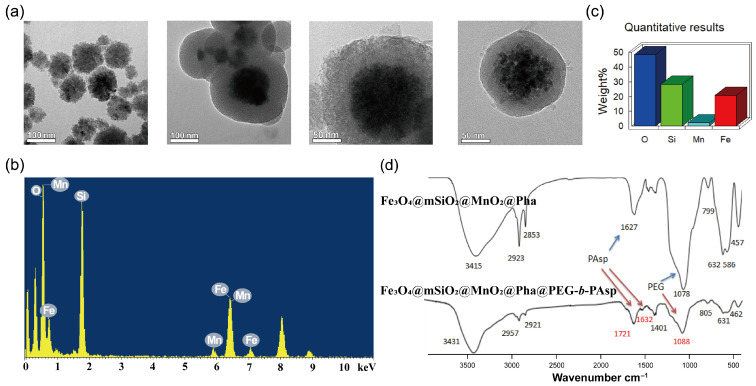
Characterization of FSM and Pha@FSMP nanoparticles. (**a**) TEM images showing morphological evolution of nanoparticles: Fe_3_O_4_ cores, Fe_3_O_4_@mSiO_2_ core-shell structures, MnO_2_-loaded FSM nanoparticles, and Pha@FSMP. (**b**) Energy dispersive X-ray spectroscopy (EDS) elemental analysis of the of FSM nanoparticles, displaying Fe, Mn, Si, and O peaks. (**c**) Quantitative elemental weight percentages of FSM nanoparticles, verifying composite fabrication. (**d**) FTIR spectra confirming successful conjugation of PEG-*b*-PAsp to FSM nanoparticles via characteristic polymer peaks.

**Figure 3:**
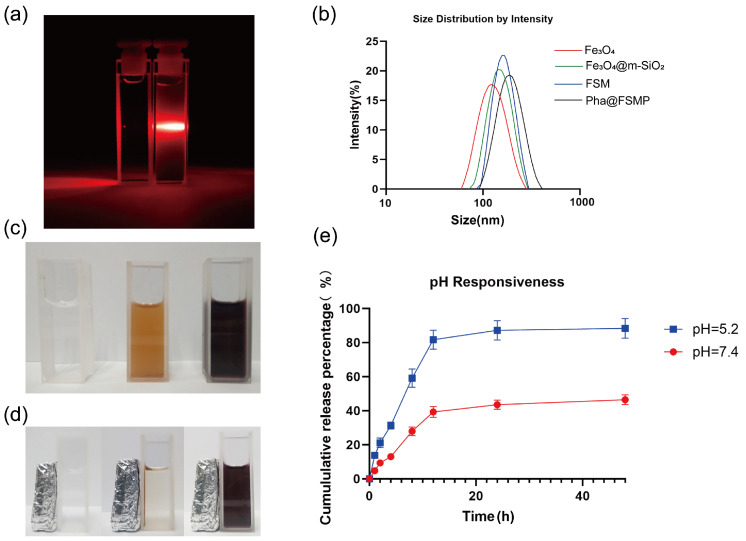
Physicochemical and functional characterization of nanoparticles. (**a**) Tyndall effect in Pha@FSMP dispersion, confirming colloidal stability. (**b**) Dynamic light scattering (DLS) showing the hydrodynamic size distribution of Fe_3_O_4_, Fe_3_O_4_@mSiO_2_, FSM, and Pha@FSMP nanoparticles. (**c**) Visual appearance of PBS (0.1 M), FSMP dispersion (50 μM), and Pha solution (50 μM). (**d**) Magnetic responsiveness of FSMP nanoparticles, showing rapid aggregation under an external magnetic field. (**e**) In vitro pH-responsive drug release profile of Pha@FSMP, with enhanced release at acidic pH (5.2) vs. physiological pH (7.4).

The microstructures of the synthesized nanoparticles were characterized by transmission electron microscopy (TEM), as shown in Figure 2a. The TEM images at different magnifications sequentially illustrate the morphological evolution during synthesis, confirming the successful preparation of Fe_3_O_4_ core nanoparticles, mesoporous silica-coated Fe_3_O_4_@mSiO_2_ nanoparticles, MnO_2_-loaded FSM nanoparticles, and Pha@FSMP. These micrographs clearly demonstrate the uniform spherical morphology, core-shell structure, and particle size distribution of the nanoparticles. TEM elemental analysis (Figure 2b) identified characteristic peaks of Fe, Mn, Si, and O elements in FSM nanoparticles. Quantitative elemental mapping (Figure 2c) further confirmed the relative weight percentages of these elements, validating the successful fabrication of the FSM composite. FTIR spectra (Figure 2d) confirmed successful conjugation of the polymer by detecting characteristic peaks at 1088 cm^−1^ (PEG backbone), 1632 cm^−1^ (P-Asp amide bond), and 1721 cm^−1^ (P-Asp carboxyl group). Compared to the free Pha solution, the Tyndall effect was observed by irradiating the Pha@FSMP dispersion with a laser beam (Figure 3a). A distinct red laser path was visible, confirming the colloidal nature and excellent hydrodynamic stability of the nanocomposites.

Dynamic light scattering (DLS) measurements (Figure 3b) revealed the hydrodynamic sizes of nanoparticles as: Fe_3_O_4_ (117.9 ± 4.89 nm), Fe_3_O_4_@mSiO_2_ (142.7 ± 5.22 nm), FSM (157.2 ± 6.60 nm), and Pha@FSMP (191.0 ± 5.73 nm), reflecting stepwise growth during synthesis.

The drug loading efficiency of Pha@FSMP was determined to be 6.09% via absorbance at 405 nm using a standard curve (Figure S1 and Table S1), representing a 10.28% improvement after synthesis optimization. Visual inspection (Figure 3c) showed distinct dispersion colors: PBS (colorless), FSMP dispersion (brown–yellow), and Pha solution (purple). Upon exposure to an external magnetic field (Figure 3d), FSMP nanoparticles rapidly aggregated near the magnet, leaving the solution clear, confirming excellent magnetic responsiveness. In vitro drug release studies (Figure 3e) demonstrated pH-dependent behavior, with cumulative release reaching ~88% at pH 5.2 vs. ~47% at pH 7.4 over 48 h, confirming robust pH-responsive drug release of the nanocomposites.

### 3.2. In Vitro Cell Experiment Results

#### 3.2.1. Cellular Uptake and Subcellular Localization

**Figure 4:**
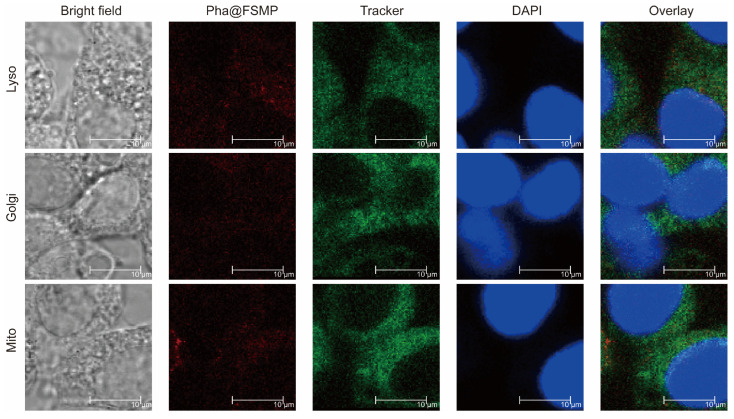
Subcellular colocalization of Pha@FSMP nanoparticles. Confocal laser scanning microscopy images showing Pha@FSMP (red) colocalization with lysosomal, Golgi, and mitochondrial trackers (green). Nuclei were stained with DAPI (blue). Overlay images confirm predominant accumulation of Pha@FSMP in lysosomes, with minimal localization in mitochondria and Golgi apparatus.

Cellular uptake efficiency was evaluated by red fluorescence intensity of Pha@FSMP (Figure 4). The strong red fluorescence signal in the Pha@FSMP group confirmed that the nanocomposite significantly enhanced cellular internalization of Pha compared to free Pha. Subcellular colocalization analyzed by confocal laser scanning microscopy (CLSM) showed that Pha@FSMP (red) predominantly colocalized with lysosomal markers (green), with minimal overlap observed in mitochondria and Golgi apparatus. This indicates that the nanoparticles were internalized via endocytosis and primarily accumulated in lysosomes, suggesting a lysosome-dependent cell death pathway.

#### 3.2.2. Cell Viability Assay

**Figure 5:**
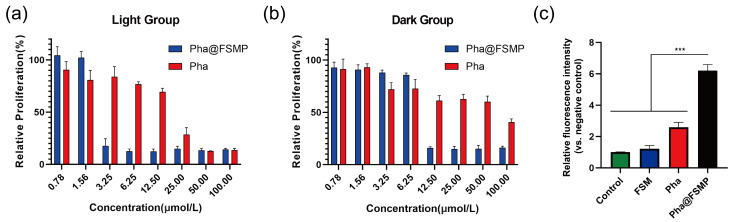
In vitro cytotoxicity and ROS generation of nanoparticles. (**a**) Antiproliferative activity of Pha@FSMP and free Pha in B16F10 cells under laser irradiation. (**b**) Cytotoxicity of formulations in the dark, confirming the light-dependent safety of Pha@FSMP. (**c**) Intracellular ROS generation in B16F10 cells at the concentration of 0.6 μg/mL, showing the highest ROS levels in the Pha@FSMP group relative to the control group (values were means ± standard deviation (SD), *n* = 3, *** *p* < 0.001).

The cytotoxicity of free Pha, FSMP, and Pha@FSMP against B16-F10 cells was evaluated using the CCK-8 assay. Under light irradiation (Figure 5a), Pha@FSMP exhibited significantly stronger antiproliferative activity than free Pha, with an IC_50_ of 2.98 μmol/L (calculated by dose–response curve analyses from GraphPad Prism 10, GraphPad Software, LLC, San Diego, CA, USA) versus ~15.42 μmol/L for free Pha, confirming the nanocomposite’s ability to enhance photodynamic therapy efficacy via targeted delivery.

In the dark control group (Figure 5b), Pha@FSMP showed minimal cytotoxicity across most tested concentrations with an IC_50_ of 8.96 μmol/L, demonstrating excellent light-dependent safety. Quantitative comparison revealed that Pha@FSMP’s cytotoxicity was ~7-fold higher than free Pha in dark (59.91 μmol/L) and ~3-fold lower than Pha@FSMP under irradiation (IC_50_ of 2.98 μmol/L), highlighting its potent tumor-killing capacity in a combining photodynamic way.

#### 3.2.3. Detection of Reactive Oxygen Species (ROS)

The photodynamic efficacy of Pha@FSMP was evaluated by quantifying intracellular relative ROS generation under laser irradiation. As shown in Figure 5c, few intracellular ROS levels were detected in the FSM group under laser irradiation, while free Pha induced a mild increase in intracellular ROS production at the concentration of 0.6 μg/mL. The Pha@FSMP treatment group at the same concentration exhibited the most robust ROS generation, with a ~6-fold increase in fluorescence intensity relative to the untreated negative control group, which was significantly higher than that of the free Pha group and FSM group (*** *p* < 0.001). This enhanced intracellular ROS production was attributed to the significant catalytic effect of MnO_2_. These results demonstrated that Pha@FSMP could effectively trigger robust intracellular ROS generation, which further correlated with its enhanced photodynamic therapeutic effect.

### 3.3. In Vivo Antitumor Effect

The in vivo antitumor efficacy of Pha@FSMP was evaluated in a B16-F10 melanoma xenograft mouse model, with mice randomized into six groups: saline control, Pha@FSMP + laser, Pha@FSMP, free Pha + laser, free Pha, and FSMP alone. No animals, experimental units, or data points were excluded from the analysis. Tumor volume, survival rate, and body weight were monitored as key endpoints. Within the parameters of this study, no evident side effects were identified.

**Figure 6:**
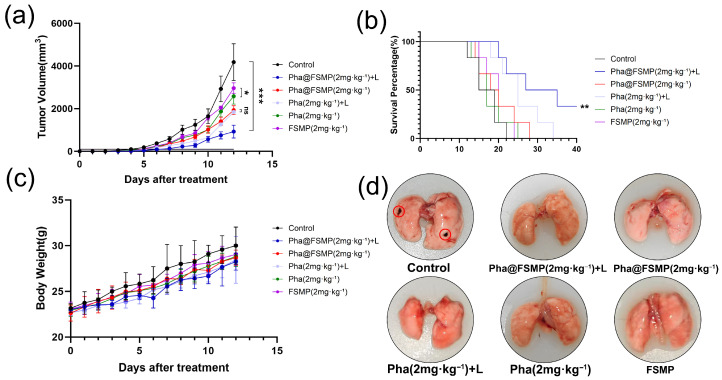
In vivo antitumor efficacy and biosafety of Pha@FSMP. (**a**) Tumor volume growth curves in B16-F10 melanoma-bearing mice, showing potent inhibition by Pha@FSMP + laser irradiation. (**b**) Kaplan–Meier survival curve demonstrating prolonged survival in the Pha@FSMP + laser group. (**c**) Body weight changes indicating minimal systemic toxicity of all formulations. (Values were means ± standard deviation (SD), *n* = 5, * *p* < 0.05, ** *p* < 0.01, *** *p* < 0.001, ns: no significance). (**d**) Macroscopic images of lung tissue, confirming absence of metastatic nodules in the Pha@FSMP + laser group. Red circles indicate the macroscopic metastatic nodules of melanoma in the lung tissue.

Under laser irradiation, the Pha@FSMP + laser group exhibited the most potent tumor growth inhibition (Figure 6a), outperforming all control groups. This enhanced efficacy is attributed to MnO_2_-mediated oxygen generation, which potentiates photodynamic therapy (PDT)—a benefit not observed with free Pha + laser or FSMP monotherapy. Survival analysis (Figure 6b) confirmed a significant survival extension in the Pha@FSMP + laser group, whereas free Pha (with or without laser) showed no improvement over the saline control, underscoring the necessity of both the nanocomposite formulation and laser irradiation for therapeutic benefit. Throughout the treatment period, no significant body weight loss was observed in any group (Figure 6c), indicating minimal systemic toxicity of the nanocomposites.

**Figure 7:**
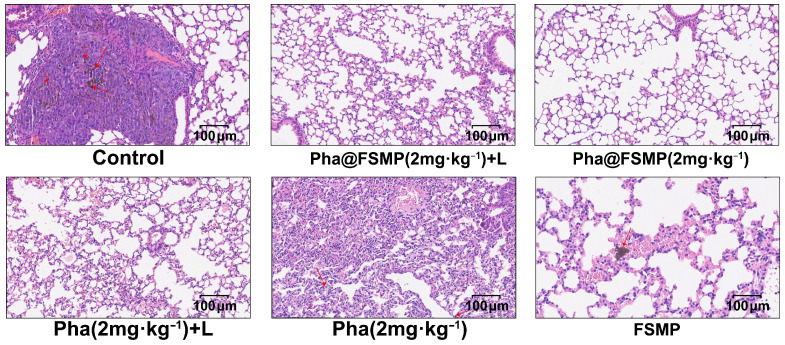
Microscopic images of the H&E stained lung sections of the C57BL/6J mice in each group verifying the anti-metastatic effect of Pha@FSMP + laser irradiation. Red arrows indicate intrapulmonary metastatic foci of malignant melanoma with characteristic melanin pigment deposition.

Given the high metastatic potential of melanoma, lung metastasis was assessed via macroscopic inspection and H&E staining. The Pha@FSMP + laser group displayed no visible metastatic nodules on lung surfaces (Figure 6d), whereas control and monotherapy groups showed extensive metastases. Histological analysis (Figure 7) further confirmed the absence of lung metastases in the Pha@FSMP + laser group, verifying its anti-metastatic activity.

Collectively, these results demonstrate that the MnO_2_-driven oxygen-elevating Pha@FSMP nanocomposite effectively suppresses melanoma growth and metastasis in vivo, highlighting its potential as a safe and potent therapeutic strategy.

## 4. Conclusions

Photodynamic therapy (PDT) remains clinically limited by the hypoxic tumor microenvironment, which impairs therapeutic efficacy. To address this critical challenge, we have engineered a multifunctional nanocomposite delivery system (Pha@FSMP) that integrates precise tumor targeting, pH-responsive drug release, and MnO_2_-mediated oxygen generation. This system not only delivers photosensitizers to tumor sites with minimal systemic toxicity but also elevates intratumoral oxygen levels, thereby significantly enhancing PDT efficacy both in vitro and in vivo. Our results demonstrate that Pha@FSMP effectively suppresses melanoma growth and metastasis, with excellent biosafety profiles observed in animal models. The underlying toxicological characteristics and pathological mechanisms were not fully explored in this study. Further studies are warranted. Collectively, this oxygen-augmented PDT platform represents a promising strategy to overcome hypoxia-associated resistance and advance clinical PDT for cancer treatment.

## Data Availability

The original contributions presented in this study are included in the article/Supplementary Material. Further inquiries can be directed to the corresponding authors.

## Supplementary Materials

The following supporting information can be downloaded at: https://www.mdpi.com/article/10.3390/pharmaceutics18050586/s1, Figure S1: The standard curve of Pha@FSMP; Table S1: Specific values of establishment of standard curves for Pha@FSMP.
