# Supplementary material for: MnO2-Incorporated Magnetic Nanoparticles with pH-Responsive Drug Release Enhance Hypoxia-Resistant Photodynamic Therapy for Melanoma
Source: Pharmaceutics. 2026 May 9;18(5):586. doi: 10.3390/pharmaceutics18050586 (PMC13210973; doi:10.3390/pharmaceutics18050586)
Supplement: Supplementary file 1 [file pharmaceutics-18-00586-s001.zip › pharmaceutics-4301904-supplementary.pdf]

Supplementary Materials

Establishment of standard curves for Pha@FSMP

The absorption value at 405nm was measured with an enzyme marker, and the average value of each group was calculated with a linear range. 0.125mg/mL~0.0005mg/mL, establishing a standard curve y = 17.515x + 0.0103, R^2 = 0.9997. The experimental results are own in Figure S1, and the specific values are shown in Table S1.

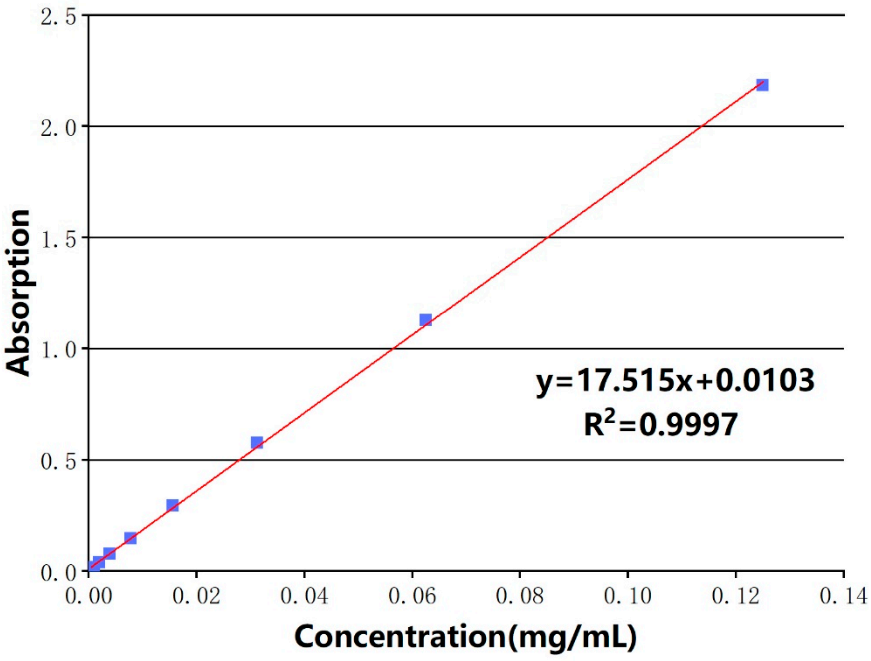

Figure S1. The standard curve of Pha@FSMP.

Table S1. Specific values of establishing standard curves for Pha@FSMP

	1	2	3	4	5	6	7	8	9
Concentration (mg/mL)	0.125	0.0625	0.03125	0.015625	0.007813	0.003906	0.001953	0.000977	0.000488
Abs	2.184333	1.125333	0.575	0.29333	0.146333	0.075667	0.037667	0.016667	0.008333
